# Restoring Large Defect of Posterior Tooth by Indirect Composite Technique: A Case Report

**DOI:** 10.3390/dj6040054

**Published:** 2018-10-07

**Authors:** Pei-Ying Lu, Yu-Chih Chiang

**Affiliations:** 1 Department of Restorative and Esthetic Dentistry, National Taiwan University, Taipei 100, Taiwan; r05422016@ntu.edu.tw; 2 Department of Restorative and Esthetic Dentistry, National Taiwan University and National Taiwan University Hospital, Taipei 100, Taiwan

**Keywords:** indirect restoration, composite onlay, dental esthetics, composite resin, large cavity

## Abstract

Advances in adhesive dentistry have led to increased use of indirect restorations. In some situations, indirect composite techniques are more advantageous than direct composite filling techniques, such as establishing proper occlusal and interproximal anatomy, reducing polymerization shrinkage stress, and promoting the degree of conversion. This article presents a case about restoring the lower right first molar with extensive loss of tooth structure by the composite onlay to achieve a proper anatomic form and rehabilitate chewing function. This one-year clinical case encourages clinicians to manage large decay of posterior tooth conservatively. The given functional and esthetic outcomes demonstrate the promising applicability of the indirect composite technique.

## 1. Introduction

Owing to the evolution of adhesive technologies and restorative materials, approaches and treatment plans for restoring posterior teeth have been considerably innovated [1]. Although amalgam and gold have demonstrated persistent clinical success and biocompatibility, novel tooth-colored restorations are increasingly replacing metal restorations not only for esthetic concerns but also for more conservative preparations [2]. Considering biology, mechanics, function, and aesthetics, a harmonious and successful restorative result could be achieved with these natural-looking restoration materials, such as resin composite and ceramic. Differences in the mechanical properties of ceramic materials and resin composites lead to the question as to which material is more durable than the other, especially in load-bearing posterior regions of the mouth. Ceramic restorations are characterized by satisfactory esthetic results and stronger physical properties. Studies on ceramic indirect restorations have revealed a success rate of approximately 90% after 10 years. However, limited information is available on the clinical survival of indirect resin composites. Previous reviews have revealed no conclusive evidence showing that indirect ceramic inlays and onlays can survive longer than resin ones in the mouth [3,4].

Although resin composites are the trends among restorative options, several issues are still associated with their properties, such as polymerization shrinkage, microleakage, marginal gap formation, color instability, difficulties in rebuilding ideal proximal contour and contact, and insufficient mechanical property [2,5,6]. The mentioned issues influence the clinical success and longevity of composites resin restorations. The rehabilitation of decayed or fractured posterior teeth through an indirect technique overcame some difficulties associated with direct composite filling, resulting in better occlusion and a desired tooth form; in addition, complete curing and reduced shrinkage of composite resins in the deepest regions can be achieved [6]. For large damaged posterior teeth, the indirect technique is indicated [7]. This report presents a case involving the restoration of an extensive cavity of the lower right first molar through an indirect composite technique and one-year follow-up of the clinical outcome afterward.

## 2. Case Presentation

**Figure 1:**
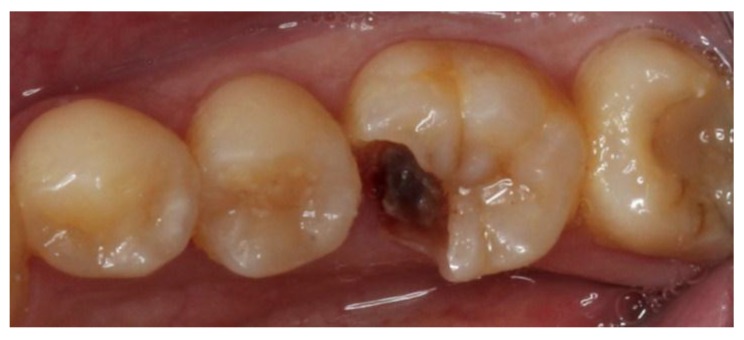
Pre-operative clinical picture of lower right first molar with large cavity over mesial side.

**Figure 2:**
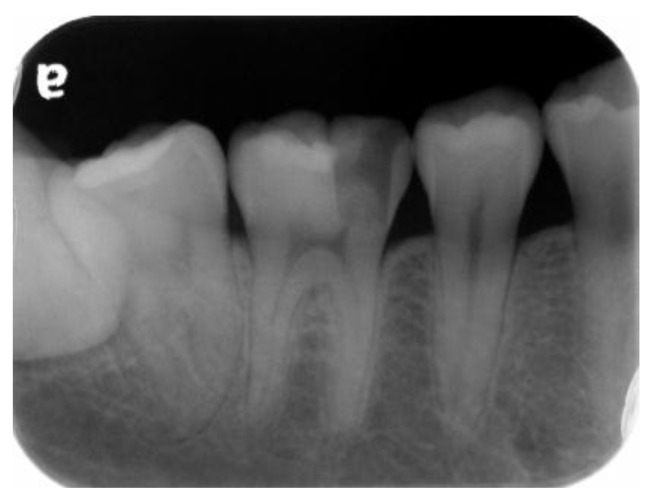
Pre-operative periapical radiograph shows caries in proximity to the pulp horn.

A 22-year-old male student visited the department of restorative and esthetic dentistry of National Taiwan University Hospital for a dislodgement of lower right posterior tooth restoration. He had received direct composite resin filling three years ago, but the old restoration was dislodged while eating popcorn two days earlier. Clinical examination revealed food impaction over the large mesial cavity of the lower right first molar with gingival inflammation (Figure 1). The pulp vitality test was normal with no symptoms or signs. Radiographic examination revealed a large mesial decay in proximity to the pulp horn while no obvious abnormal apical findings were noted (Figure 2). After oral hygiene reinforcement, we discussed with the patient that composite onlay might be a choice for his aesthetic and financial concerns, and further possibility of root canal treatment. A written informed consent was obtained before the treatment, and patient gave permission for the related pictures and radiographs to be published before submission.

**Figure 3:**
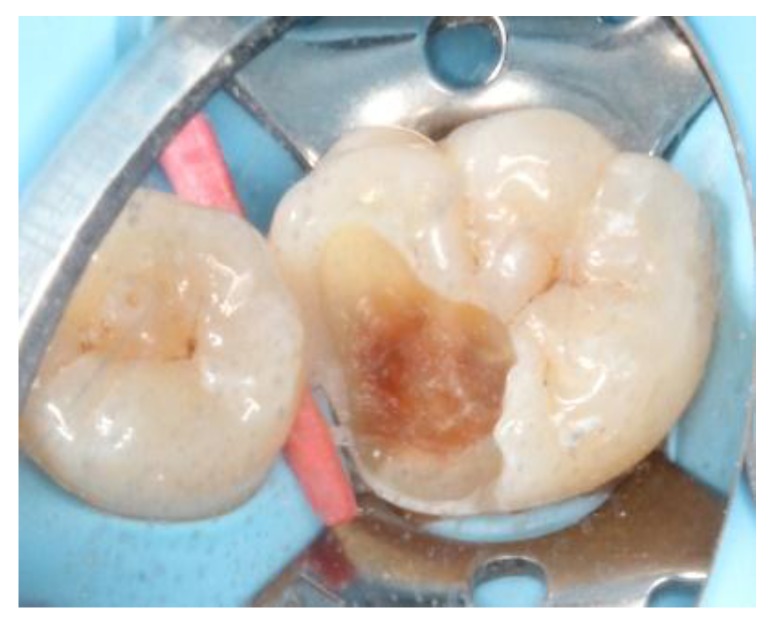
Caries removal under rubber dam isolation.

**Figure 4:**
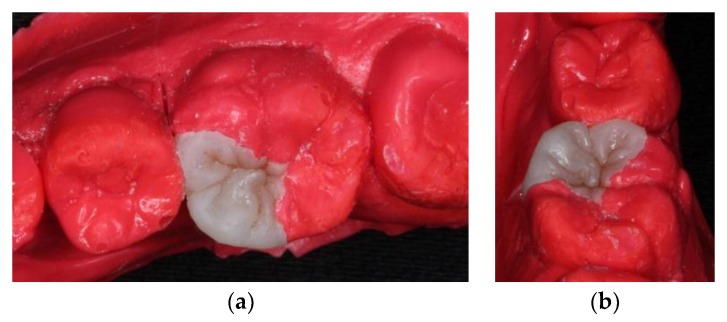
Composite onlay fabricated on silicone die: (**a**) occlusal view; and (**b**) mesial view.

At the next appointment, under block anesthesia using 2% lidocaine with epinephrine 1:100,000, caries was removed by low speed carbide burs and sharpened spoon excavator under rubber dam isolation (Figure 3). The mesial gingival margin was located approximately 0.5 mm subgingivally. Therefore, gingival displacement was achieved using a retraction cord. The undercuts of the tooth cavity were blocked out with a nano-hybrid composite resin (Grandio, shade A3, Voco, Cuxhaven, Germany), which served as a base material. The cavity was prepared, and an alginate impression was taken after removal of the rubber dam. A self-cured bis-acrylic resin (Structur 2 SC, Voco, Cuxhaven, Germany) was used as a temporary filling material before the next cementation appointment. A fast-setting silicone die material (GrandioSO Inlay System, Voco, Cuxhaven, Germany) was injected into the alginate. During the inter-appointment period, the onlay restoration was fabricated incrementally with a light-cured composite resin (Grandio, shades A2, A3, and A3.5, Cuxhaven, Germany) (Figure 4), and each layer was polymerized for 10 s with a light-emitting diode curing unit (Valo, Ultradent, South Jordan, UT, USA) with light irradiance of 1000 mW/cm^2^. For sculpting the occlusal morphology, we determined mesio-lingual cusp and trianglular ridge by referring to the existing distolingual cusp and neighboring teeth. In addition, the cast of the upper arch was used for adjusting the occlusion. The final occlusal anatomy was reconstructed and incorporated with a resin staining kit (Tetric color, Ivoclar Vivadent, Schaan, Liechtenstein), which was used for internal staining to mimic the fissures of a natural tooth. After finishing and polymerization, the composite onlay was removed from the silicone die and cured from the intaglio surface for 40 s.

**Figure 5:**
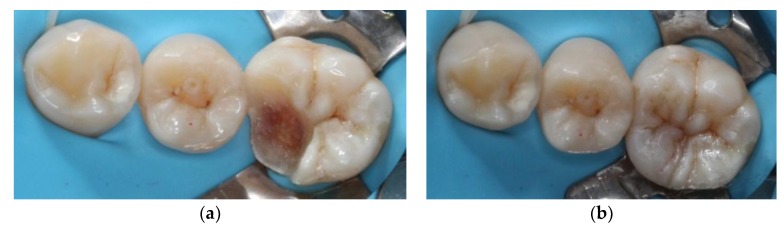
Cementation under rubber dam isolation: (**a**) before cementation; and (**b**) after cementation.

**Figure 6:**
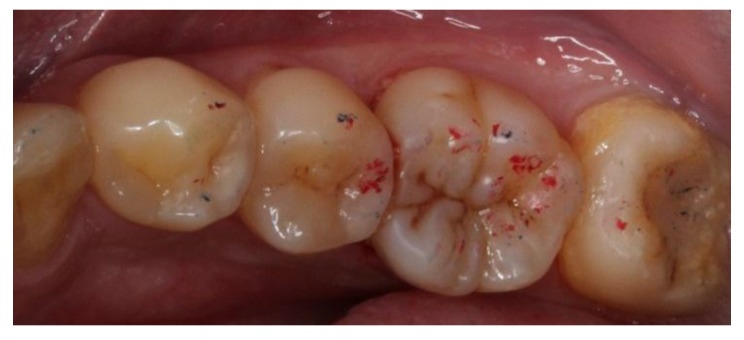
Examining the occlusal contacts after cementation. The blue spots represent the centric occlusion, and the red spots represent the functional movements.

**Figure 7:**
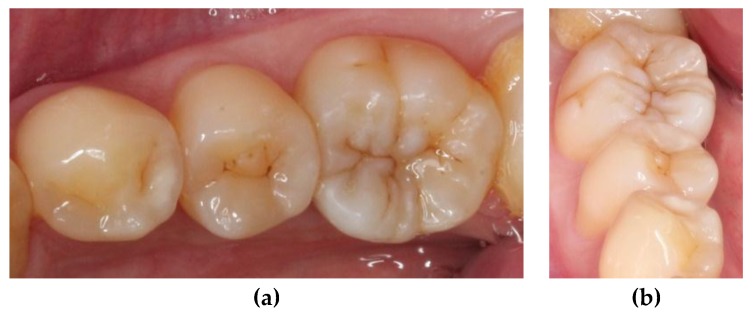
After finishing and polishing: (**a**) occlusal view; and (**b**) mesial view.

**Figure 8:**
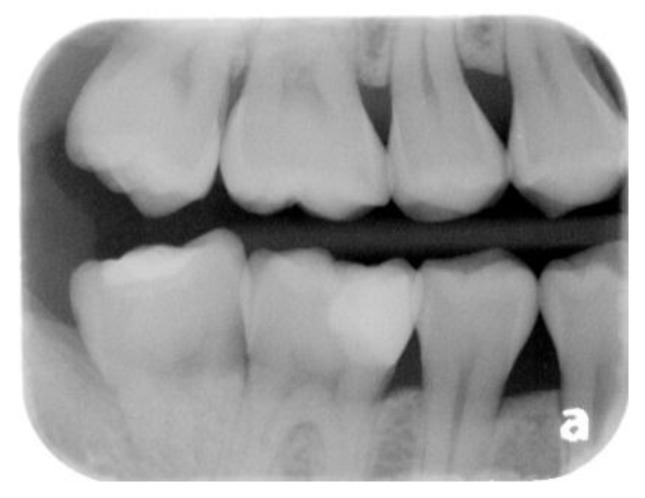
Post-operation bite-wing radiograph shows no obvious overhang or remaining excess resin cement.

**Figure 9:**
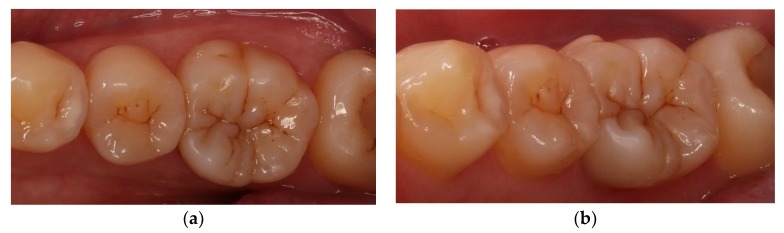
Follow-up after 12 months: (**a**) occlusal view; and (**b**) no obvious gap or marginal discoloration.

At the next appointment, isolation was performed with rubber dam. Then, the onlay was tried in and the fitness was checked. Before cementation, the intaglio surface of restoration received airborne-particle abrasion with 50 μm alumina particles; subsequently, it was conditioned with 37.5% phosphoric acid gel (Gel Etchant, Kerr, Orange, CA, USA) for 15 s. After the etchant gel was rinsed, the composite onlay was cleaned with 75% ethanol in an ultrasonic bath for 3 min. Moreover, the tooth was selectively etched with 37.5% phosphoric acid gel (Gel Etchant, Kerr, Orange, CA, USA) for 15 s, rinsed with water spray, and air dried. The self-etching adhesive and dual-cured luting composite (Multilink N system, Ivoclar Vivadent, Schaan, Liechtenstein) were used for final cementation. Polymerization was performed for 40 s per surface (Figure 5). After rubber dam removal, the occlusal contacts were adjusted and checked with articulating paper (Figure 6). Finally, the restoration was finished by fine-grained diamond burs and polished by abrasive, silicon-impregnated rubbers (Jiffy, Ultradent, South Jordan, UT, USA) (Figure 7). A bitewing radiograph was taken to examine whether any overhang existed at the gingival margin (Figure 8). At the one-year recall, the restoration still maintained its esthetic and chewing function (Figure 9).

## 3. Discussion

Interest in bonded tooth-colored restoration has been increasing in recent years. Although the color stability and wear resistance of resin composites are not as good as those of ceramic restorations, both are routinely considered by clinicians in their daily practice. Due to the chemical compositions, ceramics are harder and thus more wear resistant, but they can cause more wear than usual to the opposing tooth. In addition, ceramics are brittle and more prone to facture than composites in the case of thin-layer thickness [8]. A 4–6-year clinical study indicated that bonded indirect resin composite onlays can achieve clinical success in treating painful, cracked teeth [9]. Cusp protection of bonded indirect resin composite onlays could provide resistance to cuspal deflection regarding structurally compromised teeth [10,11]. Moreover, using resin composites is easier for intra-oral repairs in the future by composite materials. From an economic perspective, composite resins are a more attractive option than costly ceramic materials [9].

Several factors, namely the materials, adhesive cementation and bonding procedures, are relevant in applications of indirect composite restoration [3]. The resin composite used in this study was a nano-hybrid with high filler content (87% *w*/*w*). The nanohybrid composite not only provided high polishability with low surface roughness but also possibly enhanced the retention of smoothness after abrasion. Some in vitro wear studies have revealed that increased filler content enhances the wear resistance, and nanohybrid composites may exhibit superior wear resistance compared with other composite resins [10,11].

A restoration can be placed either directly or indirectly, and an indirect restoration is more favorable for restoring the morphology and function of a compromised tooth structure. The indirect technique was used outside the oral cavity, taking an impression and fabricating the composite restoration on a die model. Internal buildup by resin composites before tooth preparation can prevent excessive loss of sound tissue, which is crucial in practice because the goal of restorative dentistry is to prepare the cavity as conservatively as possible. Using a quick-setting flexible silicone die, instead of stone die, could allow a more effective treatment which could be performed on a chair side. Two recent systematic reviews regarding clinical performance with a follow-up period of at least three years revealed that no significant difference was observed between the direct and indirect methods [2,7]. The caution for such indirect composite restoration is the cementation procedure. The luting cement may adhere to the composite onlay, which cannot easily be detached from the composite surface after light cured. Occaisionally, we may encounter the challenge of occlusal adjustment. Thus, as a reference by a study cast of opposing teeth is also indicated. Nevertheless, the indirect method could reduce the time of intraoral adjustment and overcome some difficulties of the direct method, especially when dealing with severely destroyed posterior tooth or with multiple cavities at the same visit. Moreover, this technique was favored over the direct technique because it could easily reconstruct the anatomy of tooth structure and enable a more appropriate occlusal and interproximal contour [12]. Optimal curing from all directions and outside the oral cavity can also improve the degree of conversion and then enhance physical and mechanical properties [13]. Furthermore, the indirect method might reduce the polymerization shrinkage stress, resulting in a relatively ideal marginal adaption and reduced microleakage, which are the main factors responsible for the occurrence of secondary caries [12,13,14].

Several techniques have been suggested for treating the internal surface of an indirect composite restoration to increase bond strength; examples of such techniques include acid-etching, air-abrasion, silane coupling, tribochemical coating, and laser treatment [5]. Previous research showed that the effect of surface treatment on the bond strength is dependent on the different compositions and aging of the composites [15]. Hummel et al. discovered that airborne-particle abrasion with 50-μm alumina oxide followed by conditioning with phosphoric acid produced a more irregular surface and significantly greater bond strength of a hybrid composite [16]. 

One of the main purposes of reproducing the natural morphology of posterior tooth is to enhance the mastication function. The restorations must fit into the pre-existing occlusal scheme, where grinding paths are established. This can ensure that iatrogenic interference does not occur because of dental treatment [17]. At the one-year recall in the case reported herein, the restoration could potentially withstand physiological chewing force without fracture, debonding, or marginal discoloration. Although the observation time was limited to only 12 months, this technique showed satisfactory clinical performance; accordingly, it can enable dental clinicians to restore the tooth to functional and esthetic outcomes. Further research, for example on clinical performances of ceramic onlay and composite onlay, is encouraged. 

## 4. Conclusions

Improvements in adhesive and restorative materials may enable the use of indirect composite onlays to restore an extensively damaged posterior tooth. The indirect composite technique enhances the esthetics and enables clinicians to achieve conservative tooth preparations. This technique also overcomes difficulties of the direct composite technique, such as improper occlusal and interproximal anatomy, polymerization shrinkage stress, and insufficient curing. The clinical success of this technique at the one-year recall could be attributed to adequate occlusion reconstruction, well performing adhesive systems and highly wear-resistant resin composites.
